# Predictive Modeling of a Batch Filter Mating Process

**DOI:** 10.3389/fmicb.2017.00461

**Published:** 2017-03-21

**Authors:** Akshay Malwade, Angel Nguyen, Peivand Sadat-Mousavi, Brian P. Ingalls

**Affiliations:** Department of Applied Mathematics, University of WaterlooWaterloo, ON, Canada

**Keywords:** mathematical modeling, horizontal gene transfer, plasmid conjugation, uncertainty analysis, batch processing, model comparison, sensitivity analysis, identifiability

## Abstract

Quantitative characterizations of horizontal gene transfer are needed to accurately describe gene transfer processes in natural and engineered systems. A number of approaches to the quantitative description of plasmid conjugation have appeared in the literature. In this study, we seek to extend that work, motivated by the question of whether a mathematical model can accurately predict growth and conjugation dynamics in a batch process. We used flow cytometry to make time-point observations of a filter-associated mating between two *E. coli* strains, and fit ordinary differential equation models to the data. A model comparison analysis identified the model formulation that is best supported by the data. Identifiability analysis revealed that the parameters were estimated with acceptable accuracy. The predictive power of the model was assessed by comparison with test data that demanded extrapolation from the training experiments. This study represents the first attempt to assess the quality of model predictions for plasmid conjugation. Our successful application of this approach lays a foundation for predictive modeling that can be used both in the study of natural plasmid transmission and in model-based design of engineering approaches that employ conjugation, such as plasmid-mediated bioaugmentation.

## 1. Introduction

Horizontal gene transfer by plasmid conjugation can result in rapid change in bacterial populations, as exemplified by the increased prevalence of plasmid-borne antibiotic resistance genes in response to the widespread use of antibiotics post-World War II (Davies and Davies, 2010). This capacity for rapid spread of genetic elements could be harnessed as a tool, for instance in the design of cellular computing platforms (Goñi-Moreno et al., 2013) or the modification of environmental bacterial populations through plasmid-mediated bioaugmentation (Top et al., 2002).

Efforts to quantitatively characterize conjugation processes have focused on describing the transfer rate (or “fertility”) of specific plasmids in specific conditions. Early descriptions of transfer rates consisted of reports of the ratio of plasmid-donating to plasmid-receiving cells (Watanabe, 1963; Curtiss et al., 1969) or of threshold populations below which conjugation was not observed (Anderson, 1975). A systematic approach to describing transfer rates was presented by Levin et al. (1979), who developed a population-based ordinary differential equation (ODE) model to capture the temporal dynamics of conjugation in suspended batch cultures. Follow-up work (Simonsen et al., 1990) presented a simple “end-point method” to estimate a conjugation rate from a single time-point measurement.

Subsequent projects made use of this end-point method for estimating conjugation efficiency in both suspended and attached cultures (Gordon, 1992; Duncan et al., 1995; Normander et al., 1998; Licht et al., 1999; Lilley and Bailey, 2002). The ordinary differential equation model of Levin et al. (1979) employs mass-action kinetics, which are based on an assumption of spatial homogeneity. The utility of this model for describing conjugation in spatially heterogeneous conditions was defended by Simonsen (1990), whose analysis suggested that the mass-action based model accurately captures the dynamics of attached cultures inoculated from well-mixed suspensions provided the cell density is sufficiently high.

The model developed by Levin et al. (1979) was calibrated by just two parameters: a growth rate (shared by all subpopulations) and a mass-action conjugation rate. Over the years, a number of model variants have been explored, including the following. Simonsen et al. (1990) considered allowing for (i) transition to statonary phase, (ii) segregative loss, and (iii) transitory derepression of conjugation from newly formed transconjugants (zygotic induction), but found that, for the systems they were considering, none of these features significantly improved the accuracy of their end-point estimate of transmission rate. Lundquist and Levin (1986) considered the specific effects of transitory derepression in the context of plasmid maintenance. Freter et al. (1983) developed a model to describe the behavior of cultures in continuous flow reactors (as a proxy for the mammalian gut). Clewlow et al. (1990) fit a model that incorporated Verhulst logistic growth limitations in an attempt to describe populations growing in soil microcosms. Massoudieh et al. (2010) used a partial differential equation approach to address bacterial activity on granular porous media. A stochastic differential equation approach was described by Philipsen et al. (2010).

Recently, attempts to characterize conjugation dynamics have focused on capturing the spatio-temporal dynamics of the process using individual-based models (Krone et al., 2007; Merkey et al., 2011; García and Rodríguez-Patón, 2015; Goñi-Moreno and Amos, 2015). Using simulations of an individual-based model, Zhong et al. (2012) analyzed the potential accuracy of the ODE-based end-point method (and other non-spatial metrics) for estimating transfer efficiency. They found that, as expected, spatial effects can play a major role in conjugation, but that, for sufficiently dense populations, surface-associated conjugation dynamics can be reasonably well described by non-spatial models.

As highlighted by Sørensen et al. (2005), mathematical modeling approaches are crucial for characterizing and ultimately manipulating horizontal gene transfer *in situ*. As described above, mathematical characterizations of conjugation processes have focused on quantifying the rate of plasmid transfer. In this study, we pursued the more general goal of accurately characterizing all significant aspects of a proof-of-principle batch conjugation process involving two *E. coli* strains. This project was carried out toward the long-term goal of using such models in the engineering of systems involving plasmid transfer. Consequently, our primary aim was to confirm whether an ODE model of the process could be accurately calibrated and thus provide confident predictions of system behavior. We used model comparison and uncertainty analysis to arrive at a model formulation that is well-supported by our data, and tested our final calibration against experiments that demand extrapolation from the training data. (Similar model assessment techniques were employed in Philipsen et al. (2010), but the goal in that study was to accurately estimate the noise model.) Our approach is facilitated by the use of flow cytometry to assay culture subpopulations. As confirmed by del Campo et al. (2012), cytometric measurements agree with the more traditional colony counting approach, and provide improved precision without loss of accuracy.

## 2. Materials and methods

### 2.1. Laboratory experiments

#### 2.1.1. Strains, plamids, and media

All experiments were carried out using *Escherichia coli* strains DH5α and CSH26. A CSH26 strain bearing the self-conjugative plasmid pKJK10, which harbors tetracycline, streptomycin, kanamycin resistance and P(A1-04/03)::gfpmut3b genes, was a gift from the Sørensen lab (Sengeløv et al., 2001). A DH5α strain bearing plasmid pSB1C3, which carries chloramphenicol resistance and Plac::mRFP genes, was a gift from the University of Waterloo iGEM team (Biobrick ID J04450; parts.igem.org). Cultures were grown in Luria-Bertani (LB) broth or on LB agar at 37°C. Selective media was prepared with antibiotics in the following concentrations: chloramphenicol (Cm) 10 μg/ml; tetracycline (Tet) 10μg/ml. Phosphate buffered saline (PBS) was prepared as a 10-fold dilution of stock consisting of 40 g NaCl, 1 g KCl, 5.7 g Na_2_HPO_4_, and 1 g NaH_2_PO_4_ in 500 ml MilliQ water.

#### 2.1.2. Conjugation assays

Donor (CSH26) and recipient (DH5α) strains were inoculated in 5 ml of LB supplemented with appropriate antibiotic (DH5α in Cm, CSH26 in Tet) and grown overnight at 37°C at 250 rpm. To set up the experiment, 1 ml each of donor and recipient overnight cultures were centrifuged at 13,000 rpm for 1 min, decanted and resuspended in 1 ml of fresh LB broth (non-selective). Then, 400 μl of each resuspension was inoculated in 9.6 ml of fresh LB and grown in a 250 rpm shaking incubator at 37°C to an optical density (OD_600_) of 0.5 (Genesys 20 Visible Spectrophotometer, Thermo Fisher Scientific).

Filter matings were carried out in 47 mm diameter petri plates (Fisher Scientific) containing 1.5% LB agar upon which were laid 47 mm diameter 0.4 μm polycarbonate filters (Fisher Scientific). Eight filter mating experiments were carried out, numbered 1 through 8 below. In each case, donor and recipient suspensions were diluted to achieve desired cell densities prior to loading.

For experiments #1–4 the donor and recipient cultures were loaded nearly simultaneously. To begin, 200 μl of recipient cell suspension were manually spread evenly on each filter. The filters were then dried for 1 min before loading and spreading 200 μl of donor cell suspension. Plates were then kept at 37°C until the desired time-point (specified below). Experiments #5–8 followed a staggered loading protocol; the procedure was identical to experiments #1–4 except that the second suspension (donor or recipient as specified in the caption of **Figure 3**) was loaded 120 min after the first suspension was loaded.

To begin each time-point measurement, the filters were removed from the plates and suspended in PBS. Suspension volumes were chosen to account for increasing cell populations over time, at ten time-points as follows [Time (min), PBS Volume (ml)]: (0, 2), (40, 2), (80, 2), (120, 2), (160, 2), (200, 5), (240, 10), (280, 20), (320, 25), (360, 30). Suspended filters were vortexed for 2 min after which the filter was removed from the suspension. The OD_600_ of the PBS-suspended culture was recorded and the samples were analyzed by flow cytometry, as described next. Observations were made in triplicate (biological replicates—separate plates) at each time-point.

#### 2.1.3. Flow cytometric analysis

**Figure 1:**
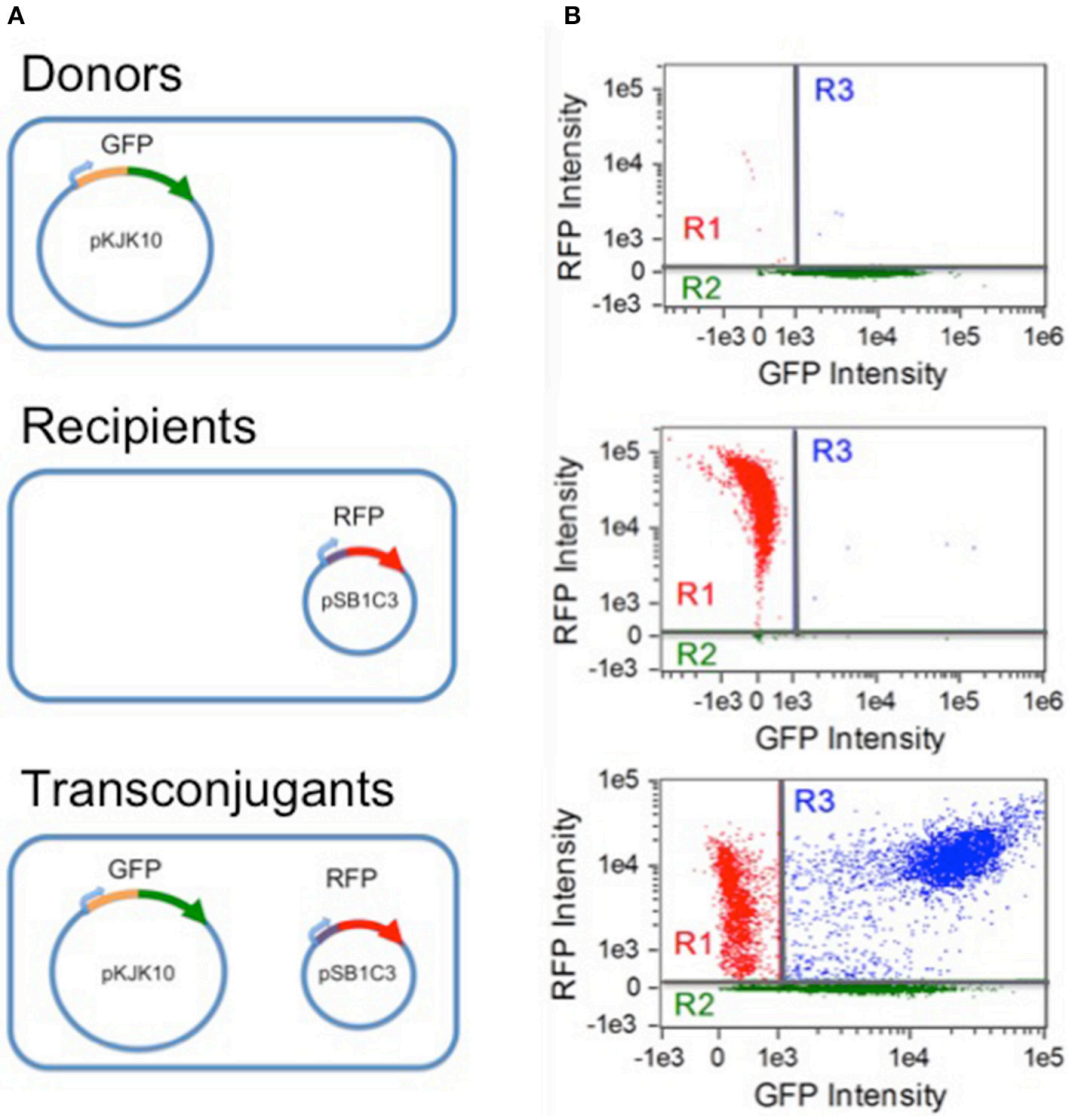
**Populations and flow cytometry gates. (A)** Three populations were involved in the experiments: donor CSH26::pKJK10 cells (GFP+), recipient DH5α::pSB1C3 cells (RFP+), and transconjugant DH5α cells harboring both plasmids (RFP+/GFP+). **(B)** Single-population controls were used to establish green and red fluorescence thresholds for identifying plasmid-bearing cells (regions R2 and R1, respectively). During the mating experiments, a population of RFP+/GFP+ transconjugants was established (region R3). The data is compensated (see Methods) and is plotted on a bi-exponential (logicle) scale (Tung et al., 2007), linear in the range [−1000, 1000].

Analysis was carried out on an Amnis Imagestream MkII flow cytometer (EMD Millipore). Data acquisition was implemented with the Amnis Inspire software package (EMD millipore). All samples were gated against gradient root mean square in the brightfield channel (labeled as gate R0), accepting images with scores between 50 and 80. Fluorescence was excited by a 488 nm solid state laser at 45 mW intensity. Green fluorescence was detected by a 533/55 nm band pass filter. Red fluorescence was detected at 610/30 nm. As a negative control, separate CSH26 and DH5α cultures (expressing GFP+/RFP- and GFP-/RFP+, respectively) were analyzed. For each, 10,000 R0 samples were collected; the results were used to construct a compensation matrix with the Amnis IDEAS software package (EMD Millipore). Gates for the three subpopulations of interest were then defined by setting thresholds for compensated non-fluorescence in the red and green channels, as shown in Figure 1. Cells were taken to be RFP- (gate R2) if the red fluorescence intensity was below 256.63. RFP+ cells were considered GFP- if the green fluorescence intensity was below 1,036.2 (gate R1). At each experimental time point, for each replicate, 20,000 R0 events were collected and sorted into the three subpopulations based on the threshold gating.

#### 2.1.4. Estimating population sizes

For each measurement, the flow cytometry results indicate the population distribution among three subpopulations: donors (GFP+/RFP−), recipients (GFP−/RFP+), and transconjugants (GFP+/RFP+). Together with the OD_600_ readings, these give relative measures of population size which were used to calibrate and analyse the model. To estimate corresponding absolute population sizes, we made use of the rule-of-thumb scaling for *E. coli* of 1.0 OD_600_ to 8 × 10^8^ cells per ml in LB broth (www.genomics.agilent.com/biocalculators/calcODBacterial.jsp, see also Milo and Phillips, 2015). To do so, we determined a scaling between OD_600_ in PBS and OD_600_ in LB. Using cultures from preliminary filter mating experiments with OD_600_ in PBS ranging from 0.073 to 1.20, we resuspended and measured OD_600_ in LB. Thirty-six samples (data not shown) were fit, resulting in the relation (OD_600_[LB]) = 0.869 × (OD_600_[PBS]) - 0.0057, with *R*^2^ of 0.995. Finally, to estimate populations on the filters, we assayed the efficiency of cell recovery by loading filters as described above and then comparing the OD_600_ of the recovered population with the original suspension; these tests (data not shown) indicated a recovery efficiency of about 50%. Consequently, to estimate the cell counts on the filter, we multiplied our cell counts in suspension by a factor of two after accounting for the corresponding suspension volume to yield a cell count per ml. Finally, we divided by the filter area (17.35 cm^2^) to arrive at a measure of cell density (cells/cm^2^).

### 2.2. Mathematical modeling

#### 2.2.1. Simulation and calibration

Models were simulated with the ode23s numerical integration routine in MATLAB (Mathworks). As described above, triplicate observations were made of the three culture subpopulations over a range of time points. For each model variant considered, and for each parameterization p, model predictions were compared against the training data (experiments #1–4) via the weighted sum of squares function:

(1) $$\begin{array}{r} \text{SSE}\left(\text{p}\right)=\underset{i}{\sum }\underset{k}{\sum }\frac{{\left(y_{obs}^{i}\left(t_{k}\right)-y_{sim}^{i}\left(\text{p},t_{k}\right)\right)}^{2}}{{\left(\sigma^{i}\left(t_{k}\right)\right)}^{2}} \end{array}$$

where $y_{obs}^{i}\left(t_{k}\right)$ is the mean of the replicate measurements of subpopulation *i* at time *t*_*k*_, $\sigma^{i}\left(t_{k}\right)$ is the standard deviation of those measurements, and $y_{sim}^{i}\left(\text{p},t_{k}\right)$ is the model prediction of subpopulation *i* at time *t*_*k*_. Here *i* runs over the four training experiments (#1–4) and the three subpopulations (donors, recipients, transconjugants), while *t*_*k*_ runs over the time-points: 0, 40, 80, 120, 160, 200, 240, 280, 320, 360 min (from initial loading of the filter).

For each model variant, the SSE was minimized by application of global optimization routines (simulated annealing, MATLAB function simannealbnd, and interior point algorithm, MATLAB function fmincon). Multiple starting points were employed to improve the chance of finding the global minimum. Parameter values were bound to the range [0, 500]. The initial values of the donor and recipient populations were treated as free parameters, to avoid over-weighting the measurements at these time-points. Transconjugant populations were initialized to zero.

#### 2.2.2. Model comparison

We used the Akaike Information Criterion (AIC) (Akaike, 1974; Burnham et al., 2011) to assess the suitability of each model. AIC describes a trade-off between the quality of the fit and the number of degrees of freedom granted by the model formulation. The corrected form of AIC was used to guard against overfitting:

(2) $$\begin{array}{r} \text{AICc}=2m+n\;\ln\;\left(\frac{\text{SSE}}{n}\right)+\frac{2m\left(m+1\right)}{n-m-1} \end{array}$$

where *m* is the number of model parameters (kinetic parameters plus eight free initial conditions: donors and recipients in each of four experiments), *n* = 116 is the number of observations (four experiments, ten time-points, three populations, less the four initial transconjugant populations assumed to be zero), and SSE is the minimal weighted sum of squared errors (Equation 1). Presuming the errors are independent and normally distributed, the AICc provides a relative measure of the strength of evidence for each model; smaller AICc values correspond to increased model suitability.

#### 2.2.3. Uncertainty analysis

Uncertainty analysis was applied to gauge confidence in the best-fit estimates of the kinetic parameters. The initial conditions were not included in this analysis, as model-based predictions will not rely on estimates of initial conditions.

##### 2.2.3.1. Sensitivity coefficients

Local absolute sensitivity coefficients were defined as:

(3) $$\begin{array}{r} S_{i,j}\left(t_{k}\right)=\frac{\partial y_{sim}^{i}\left(\text{p},t_{k}\right)}{\partial p_{j}}{|}_{t=t_{k}} \end{array}$$

where $y_{sim}^{i}\left(\text{p},t_{k}\right)$ is the *i*-th model output at time-point *t*_*k*_ and *p*_*j*_ is the *j*-th parameter. These derivatives were approximated by finite differences of 1% in *p*_*j*_. Absolute sensitivity coefficients were scaled to generate dimensionless relative sensitivity measures:

$$\begin{array}{l} {\tilde{S}}_{i,j}\left(t_{k}\right)=\frac{p_{j}}{y_{sim}^{i}\left(\text{p},t_{k}\right)}S_{i,j}\left(t_{k}\right). \end{array}$$

The overall sensitivity measure:

(4) $$\begin{array}{c} {\tilde{S}}_{j}=\sqrt{\underset{k}{\sum^{\text{​}}}\underset{i}{\sum^{\text{​}}}{\left({\tilde{S}}_{i,j}\left(t_{k}\right)\right)}^{2}} \end{array}$$

where *i* runs over all subpopulations and experiments, and *t*_*k*_ runs over all time-points, describes the degree to which each model parameter *p*_*j*_ influences the model outputs.

##### 2.2.3.2. Identifiability scores

To account for correlation among the parameters, the orthogonalization approach of Yao et al. (2003) was applied to arrive at practical measures of identifiability, as follows. A sensitivity coefficient matrix was constructed by arranging the relative sensitivity coefficients for each parameter column-wise:

(5) $$\begin{array}{r} \tilde{\text{S}}=\left[\begin{array}{ccc} {\tilde{S}}_{1,1}\left(t_{1}\right) & \cdots & {\tilde{S}}_{1,n_{p}}\left(t_{1}\right)\\ \vdots & \ddots & \vdots \\ {\tilde{S}}_{n_{o},1}\left(t_{1}\right) & \cdots & {\tilde{S}}_{n_{o},n_{p}}\left(t_{1}\right)\\ {\tilde{S}}_{1,1}\left(t_{2}\right) & \cdots & {\tilde{S}}_{1,n_{p}}\left(t_{2}\right)\\ \vdots & \ddots & \vdots \\ {\tilde{S}}_{n_{o},1}\left(t_{n_{T}}\right) & \cdots & {\tilde{S}}_{n_{o},n_{p}}\left(t_{n_{T}}\right) \end{array}\right] \end{array}$$

where there are *n*_*p*_ parameters, *n*_*o*_ observables (running over experiments and subpopulations), and *n*_*T*_ time-points. The column with the largest 2-norm (square root of sum of squared entries) is labeled **X**_1_. The corresponding parameter is judged the most identifiable; its identifiability score is the 2-norm of the corresponding column. Each column of $\tilde{\text{S}}$ is then projected onto **X**_1_, and the residuals are collected in matrix **R**_2_:

(6) $$\begin{array}{r} {\text{R}}_{2}=\tilde{\text{S}}-{\text{X}}_{1}{\left({\text{X}}_{1}^{T}{\text{X}}_{1}\right)}^{-1}{\text{X}}_{1}^{T}\tilde{\text{S}}. \end{array}$$

The column of **R**_2_ with the largest 2-norm corresponds to the next most identifiable parameter. The matrix **X**_1_ is then concatenated with the column of $\tilde{\text{S}}$ that corresponds to that parameter, to form matrix **X**_2_. The residuals of the projection of $\tilde{\text{S}}$ onto **X**_2_ are then determined (organized into matrix **R**_3_), and the third most identifiable parameter is identified. This process is iterated to provide an identifiability score for each parameter (i.e., the 2-norm of the corresponding column vector of matrix **R**_*j*_).

##### 2.2.3.3. Confidence intervals

Two complementary approaches were applied. Both begin with construction of a matrix **S** as in Equation (5) but using the *absolute* (unscaled) sensitivity coefficients *S*_*i,j*_ (Equation 3) as entries. Assuming the experimental errors are independent and normally distributed, the least squares error in Equation (1) can be used to provide a lower bound on the radius of the 95% confidence interval for parameter *p*_*j*_ of Ashyraliyev et al. (2009):

(7) $$\begin{array}{r} \Delta_{p_{j}}=\frac{m}{n-m}\text{SSE}\left(\hat{\text{p}}\right)F_{0.05}\left(m,n-m\right){\left(\sqrt{{\left({\text{S}}^{T}\left(\hat{\text{p}}\right)\text{S}\left(\hat{\text{p}}\right)\right)}_{jj}}\right)}^{-1} \end{array}$$

where $\hat{\text{p}}$ is the parameter estimate (i.e., the minimizer of SSE(p)), *m* is the number of parameters, *n* = 108 is the number of observations (four experiments, three subpopulations, nine non-initial time-points), *F*_0.05_(*m,n* − *m*) is the value at 0.95 of the inverse of the cumulative distribution function for the Fisher's distribution with *m* and *n* − *m* degrees of freedom, and (·)_*jj*_ is the *jj*-th matrix element. These results are reported as relative estimates ($\Delta_{p_{j}}/{\hat{p}}_{j}\times 100\%$) in the following section.

A complementary approach to estimating confidence intervals, following (Emery and Nenarokomov, 1998; Gadkar et al., 2005), involves constructing the Fisher Information Matrix:

$$\begin{array}{l} \text{FIM}={\text{S}}^{T}\text{W}\text{S} \end{array}$$

where **W** is the inverse of the measurement covariance matrix. Then assuming the measurement errors are independent and normally distributed, a lower bound on the radius of the 95% confidence interval for parameter *p*_*j*_ is given by:

(8) $$\begin{array}{r} \Delta_{p_{j}}^{2}=1.96\sqrt{{\left({\text{FIM}}^{-1}\right)}_{jj}} \end{array}$$

Given the samples sizes available in this study (triplicate observations), we could not estimate the full measurement covariance matrix. The measurement variances (diagonal entries in **W**) were calculated for each observation; the covariances (off-diagonal terms) were set to zero. Again, the relative estimate is reported.

## 3. Results

### 3.1. Model development and model comparison

Model development began with the three-state model of Levin et al. (1979) that describes dynamics of a recipient population (*R*), a donor population (*D*), and a transconjugant population (*T*). The model dynamics depend on the following assumptions: (1) the conjugation rate is jointly proportional to the abundance of plasmid-bearing and plasmid-free cells, (2) plasmid loss by segregation is negligible, (3) newly formed transconjugants can donate plasmids immediately, (4) the original donors and the transconjugants transmit plasmids at the same rate, (5) all subpopulations grow at the same exponential rate. The model takes the form:

(9) $$\begin{array}{r} \frac{d}{dt}D\left(t\right)=\psi D\left(t\right)\\ \frac{d}{dt}R\left(t\right)=\psi R\left(t\right)-\gamma R\left(t\right)\left(D\left(t\right)+T\left(t\right)\right)\\ \frac{d}{dt}T\left(t\right)=\psi T\left(t\right)+\gamma R\left(t\right)\left(D\left(t\right)+T\left(t\right)\right) \end{array}$$

where ψ is the growth rate and γ is the conjugation rate. This model captures the essential features of conjugation dynamics, but a more complex model might be needed for accurate prediction of mating dynamics in specific conditions; in particular, we wanted to allow for distinct growth rates and growth profiles for all subpopulations. Consequently, we considered model extensions that incorporate (i) growth lag, as in Baranyi et al. (1993), (ii) transition to stationary phase, as in Simonsen et al. (1990), and (iii) distinct transmission and growth kinetics for each subpopulation. The most general model formulation we considered involves the three subpopulations (measured in cells/cm^2^) and the fractional abundance of a limiting resource *C* (dimensionless measure, with initial value one):

(10) $$\begin{array}{l} \frac{d}{dt}D(t)=\psi_{D}(t,C(t))D(t)\\ \frac{d}{dt}R(t)=\psi_{R}(t,C(t))R(t)-R(t)(\gamma_{D}(C(t))D(t)+\gamma_{T}(C(t))T(t))\\ \frac{d}{dt}T(t)=\psi_{T}(t,C(t))T(t)+R(t)(\gamma_{D}(C(t))D(t)+\gamma_{T}(C(t))T(t))\\ \frac{d}{dt}C(t)=-e_{D}\psi_{D}(t,\;C(t))D(t)-e_{R}\psi_{R}(t,\;C(t))R(t)-e_{T}\psi_{T}(t,\;C(t))T(t) \end{array}$$

where, for each index *i* = *D*, *R*, *T*, the parameter *e*_*i*_ is a measure of resource depletion (cell^−1^ cm^2^), and the growth rates ψ_*i*_ and plasmid transfer rates γ_*j*_ (*j* = *D*, *T*) are given by:

$$\begin{array}{l} \psi_{i}\left(t,C\right)=\psi_{i,\text{max}}\left(\frac{t^{n_{i}}}{K_{L,i}^{n_{i}}+t^{n_{i}}}\right)\left(\frac{C}{K_{G,i}+C}\right)\\ \gamma_{j}\left(C\right)=\gamma_{j,\text{max}}\frac{C}{K_{T,j}+C} \end{array}$$

with ψ_*i*,max_ (min^−1^) and γ_*j*,max_ (min^−1^ cell^−1^ cm^2^) the maximal growth and transfer rates, respectively, *K*_*G,i*_ and *K*_*T,j*_ the resource levels at which growth and transfer are half maximal (dimensionless Monod constants), *K*_*L,i*_ the time at which the growth rate is half maximal (min), and the Hill coefficient *n*_*i*_ characterizing the abruptness of the end of lag phase (dimensionless).

This fully general model involves nineteen kinetic parameters, compared to just two for the original model of Levin et al. (1979) (Equation 9). We expected that a suitable model could be found between these two extremes. To identify the most suitable formulation, we applied the Akaike information criterion, as described in the Methods section, to a range of model variants. Each model variant was fit against the training data as described in the Methods section (116 triplicate data points collected from experiments #1–4). For each experiment, the initial abundance of recipient and donor subpopulations were treated as free variables, to avoid overweighting the measurements at these time-points. Thus, each model fit addressed these eight free variables in addition to the free kinetic parameters. The model comparison results are shown in Table 1; the model variants are described below and in the Table caption.

**Table 1:** **Model comparison**.

**Variant**	**Kinetic parameters**	**Free parameters**	**SSE**	**AICc**
V0	19	27	416.1	219.3
V1	16	24	420.7	210.6
V2	11	19	420.7	195.4
V3	10	18	420.7	192.5
V4	9	17	495.5	208.7
V5	9	17	420.7	189.7
V6	8	16	420.8	187.0
V7	7	15	420.8	184.3
V8	6	14	471.1	194.7
V9	6	14	514.8	205.0
V10	6	14	514.9	205.0
V11	6	14	577.0	218.2
V12	6	14	490.1	199.3
V13	4	12	651.8	227.3
V14	2	10	700.1	230.6
V15	6	14	420.8	181.6

*Model variants. V0 is Equation (10). V1 is V0 with all n_i_ = 1. V2 is V1 in the limit of large K_G,i_, K_T,j_ (i.e., growth rates are proportional to C). V3 is V2 with e_T_ = e_R_. V4 is V2 with e_T_ = e_R_ = e_D_. V5 is V3 with K_L,t_ = K_L,r_. V6 is V3 with K_L,t_ = K_L,r_ = K_L,d_. V7 is V6 with γ_T, max_ = γ_D, max_. V8 is V7 with e_T_ = e_R_ = e_D_. V9 is V7 with K_L,t_ = K_L,r_ = K_L,d_ = 0. V10 is V7 with ψ_T, max_ = ψ_R, max_. V11 is V7 with ψ_R, max_ = ψ_D, max_. V12 is V7 with ψ_T, max_ = ψ_D, max_. V13 is the model in Simonsen et al. (1990). V14 is Equation (9). V15 is V7 with ψ_T, max_ = 0*.

The full model (Equation 10), labeled as model variant 0 (V0), resulted, of course, in the best fit (lowest SSE), but at the cost of a high degree of parameterization, as indicated by a high AICc value. An exhaustive comparison of all model formulations (which, through combinatorial combination would number in the thousands) was not feasible. Instead, we carried out a strategic comparison of a targeted set of model variants, as follows.

Our first steps toward model reduction were motivated by the parameter fits. Estimates of *n*_*i*_ were close to one, while those of *K*_*G,i*_ and *K*_*T,j*_ were large relative to one. Eliminating these parameters led to models V1 and V2, with a modest reduction in quality of fit but a significant improvement in AICc score. Next, our expectations of system behavior led us to consider model V3, in which the resource-depletion rates of recipients and transconjugants are constrained to be equal, and V4, in which all three resource-depletion rates are equal. Likewise, models V5 and V6 incorporate assumptions of shared growth lag. Next, model V7 was reached by constraining the transmission rates of donors and transconjugants to be equal (reflecting an absence of transitory derepression); this simplification was motivated by similarity in the values of the corresponding parameter estimates.

As expected, each reduction in model complexity corresponds to a reduction in the quality of fit (i.e., larger SSE). The AICc values in Table 1 indicate that, for some simplifications, the loss of accuracy was compensated by reduction in degrees of freedom; for others, it was not. In particular, model V7 has the lowest AICc value of the first eight variants.

Models V8 through V12 represent further reductions of model V7: constraining all resource-depletion rates to be identical (V8); constraining all lag times to be zero (V9); constraining pairs of growth rates to be identical (V10, V11, V12). In each case the AICc indicates that the loss in accuracy is not compensated by the reduction in model complexity.

At this point, we did not expect to achieve an improved AICc value by further model reduction. Nevertheless, for completeness, we carried out comparisons with the model of Simonsen et al. (1990) (V13, with four kinetic parameters—common growth, transmission, resource-depletion rates and Monod constant) and the model of Levin et al. (1979) (V14, with two kinetic parameters, Equation 9). The corresponding AICc values indicate that these models are less suitable than model V7.

A final variation came after a preliminary identifiability analysis indicated that, contrary to our expectations, the transconjugant growth rate could not be confidently distinguished from zero. (Indeed the estimated value of this parameter was orders of magnitude smaller than the estimated growth rates for the other two populations.) Model V15 resulted from fixing the transconjugant growth rate to zero in model V7.

**Figure 2:**
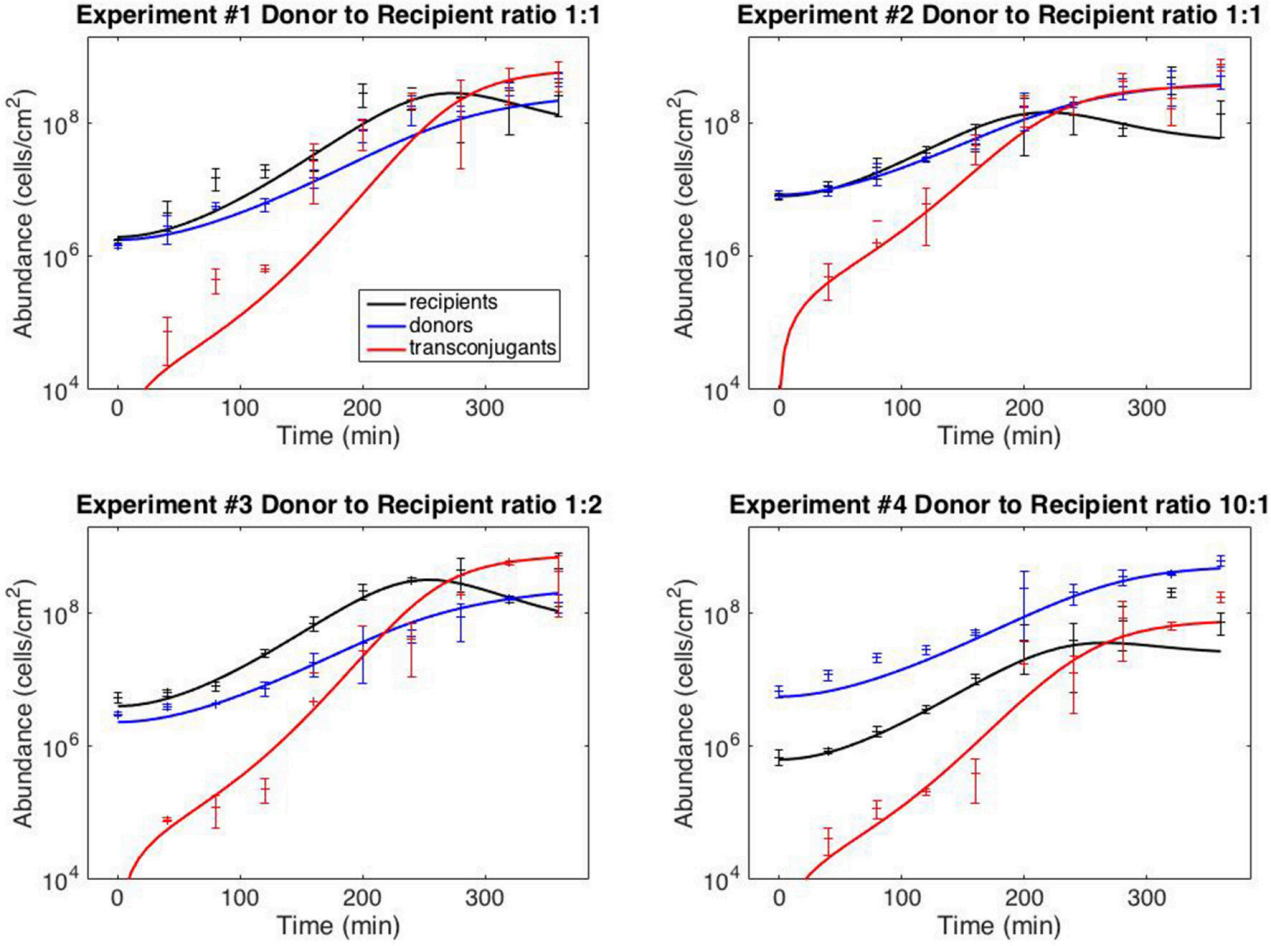
**Model fits**. Data points correspond to time-point flow cytometric and OD_600_ readings as described in Methods. Error bars correspond to standard deviation of triplicate observations. Curves are best-fit model simulations. Best-fit initial conditions (*R*_0_, *D*_0_) in units of cells/cm^−2^ are: Exp. #1: (1.96 × 10^6^; 1.77 × 10^6^); Exp. #2: (8.05 × 10^6^; 8.42 × 10^6^); Exp. #3: (4.02 × 10^6^; 2.31 × 10^6^); Exp. #4: (6.35 × 10^5^; 5.57 × 10^6^). Initial transconjugant populations are taken as zero.

The most suitable model formulation (V15) is:

(11) $$\begin{array}{l} \frac{d}{dt}R(t)=\psi_{R,\text{max}}\left(\frac{t}{K_{L}+t}\right)C(t)R(t)\\ \;-R(t)\left(\gamma_{\text{max}}C(t)\left(D(t)+T(t)\right)\right)\\ \begin{array}{c} \frac{d}{dt}D(t)=\psi_{D,\text{max}}\left(\frac{t}{K_{L}+t}\right)C(t)D(t) \end{array}\\ \frac{d}{dt}T(t)=R(t)\left(\gamma_{\text{max}}C(t)(D(t)+T(t))\right)\\ \frac{d}{dt}C(t)=-\left(\frac{t}{K_{L}+t}\right)C(t)\left(e_{R}\psi_{R,\text{max}}R(t)-e_{D}\psi_{D,\text{max}}D(t)\right) \end{array}$$

with six kinetic parameters: ψ_*R*,max_, ψ_*D*,max_, *K*_*L*_, γ_max_, *e*_*R*_ and *e*_*D*_. The model fits against experiments #1–4 are shown in Figure 2 (the full dataset is provided in Supplementary File S1); best-fit kinetic parameter values are reported in the next section.

### 3.2. Model assessment

#### 3.2.1. Uncertainty analysis

Uncertainty analysis was carried out on model V15 (equation 11) as described in the Methods section. The initial conditions were not considered free parameters in this analysis, because we are not concerned with accurately estimating the initial conditions when using the model to predict behavior. To determine the sensitivity coefficients, simulations were run from the best-fit initial conditions (reported in the caption of Figure 2). The results of the uncertainty analysis are presented in Table 2.

**Table 2:** **Uncertainty analysis**.

**Parameter**	**Best fit value**	**Sensitivity**	**Identifiability**	**95% CI FIM (%)**	**95% CI Δ (%)**
ψ_*D*,max_	0.0392 min^−1^	12.8	11.2	±8.80	±37.3
ψ_*R*,max_	0.0571 min^−1^	19.1	15.7	±20.2	±16.9
γ_max_	1.27 × 10^−10^ min^−1^ cell^−1^ cm^2^	26.2	26.2	±20.1	±11.9
*e*_*D*_	1.86 × 10^−9^ cell^−1^ cm^2^	4.51	3.09	±15.6	±65.0
*e*_*R*_	6.22 × 10^−10^ cell^−1^ cm^2^	4.14	2.36	±16.6	±66.7
*K*_*L*_	145 min	12.2	1.06	±32.5	±43.8

#### 3.2.2. Comparison to test data

**Figure 3:**
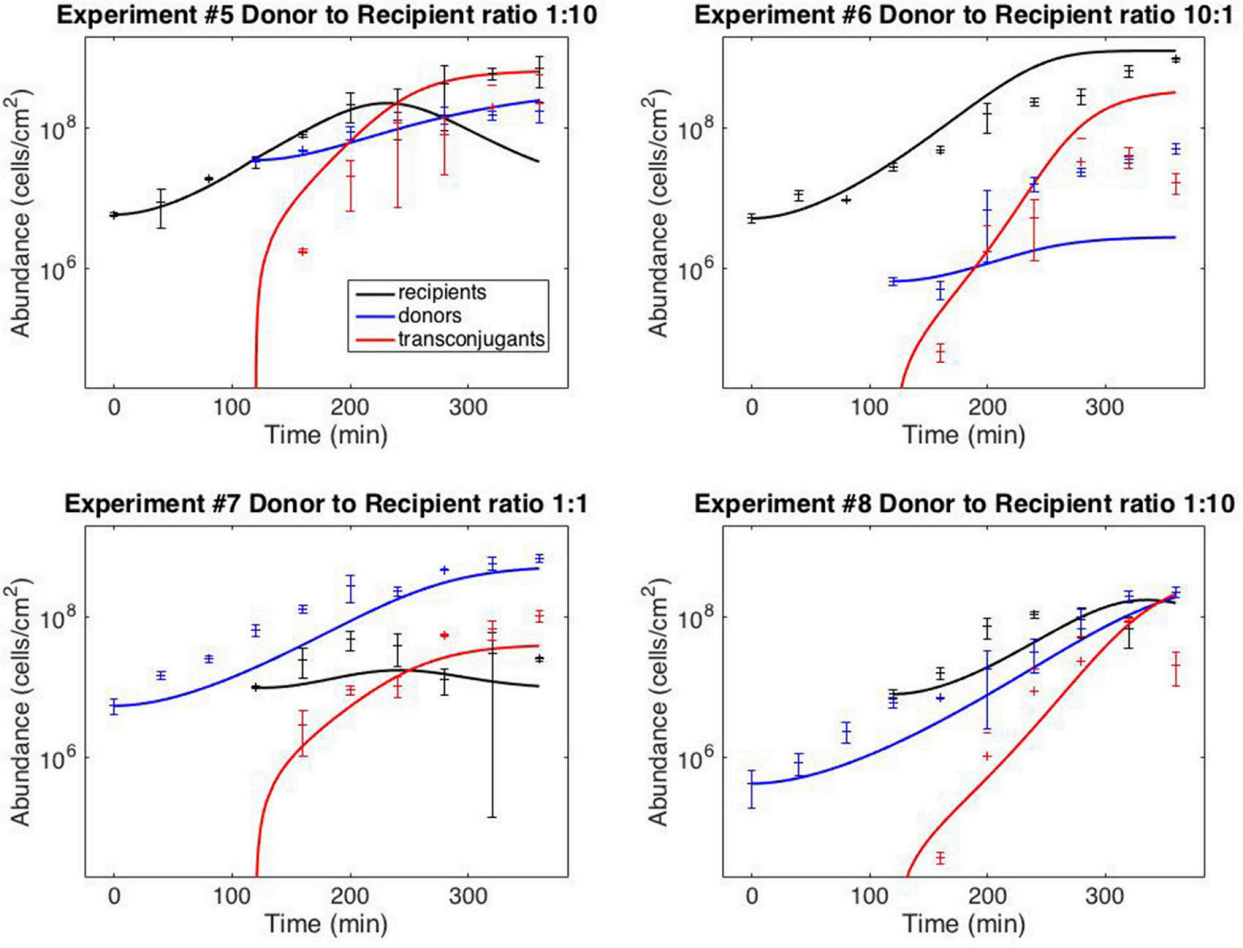
**Model predictions**. Data points correspond to time-point flow cytometric and OD_600_ readings as described in the Methods Section. Error bars correspond to standard deviations of triplicate observations. Curves are simulations of model (11). Initial conditions correspond to mean observations, as follows (in units of cells/cm^−2^): Exp. #5: *R*(0) = 5.92 × 10^6^, *D*(120) = 3.51 × 10^7^; Exp. #6: *R*(0) = 5.24 × 10^6^, *D*(120) = 6.71 × 10^5^; Exp. #7: *D*(0) = 5.51 × 10^6^, *R*(120) = 1.00 × 10^7^; Exp. #8: *D*(0) = 4.34 × 10^5^; *R*(120) = 8.07 × 10^6^. Simulations of delayed loading incorporated the delay into the growth lag (i.e., time *t* was replaced by (*t* − 120) for the populations that were loaded at *t* = 120).

Figure 3 shows the results of using model V15 (Equation 11) to predict the results of the staggered loading experiments #5–8, in which one of the two subpopulations was loaded 120 min after the first (the full dataset is provided in Supplementary File S1). For the simulations, the initial donor and recipient populations were set to the observed values. The transconjugant population was set at zero up to time 120.

## 4. Discussion

### 4.1. Model development

By comparing model variants by the Akaike information criterion (rather than relying on expectations of system behavior) we arrived at an unbiased selection of the model formulation that is best supported by the experimental results. AIC-based model comparison is technically only justified when the errors are independent and normally distributed. That condition is only approximately satisfied in this case. (The errors, not shown, are symmetric, but are more heavy-tailed then a normal distribution.) Nevertheless, the AIC provides a useful guide to model comparison. Notably, it led to a model for which (i) there is no transitory derepression of the conjugative machinery, and (ii) the newly-formed transconjugants exhibit negligible growth over the experimental time-frame. These findings represent hypotheses regarding the plasmid-recipient pair under investigation. The Sørensen lab has carried out a number of studies addressing conjugation of pKJK10's parental plasmid pKJK5 (e.g., Bahl et al., 2007a,b), but the specific dynamic features represented by the model have not been previously investigated.

The estimated growth kinetics in Table 2 must be interpreted carefully. Considered individually, the estimates correspond to unreasonable maximal doubling times of only 18 min for donors, 12 min for recipients, and a long lag phase—half maximal growth reached after 145 min. These best-fit estimates cannot be interpreted in isolation; they provide accurate descriptions of behavior in the specific context of a rapidly-depleting limiting resource. (In all simulations the growth rates were always well below their maximal values). Alternative experimental conditions (e.g., fed-batch) could allow the lag and maximal growth rates to be independently assessed. (The model variants that do not include lag or resource limitation provide some indication of what might be expected; e.g., for model V14, Equation 9) the common doubling time is estimated to be about 50 min).

### 4.2. Model assessment

The overall sensitivity measure in Table 2 is a relative score indicating the degree to which the observations are influenced by each parameter; small scores indicate that the available data may be insufficient to accurately constrain parameter estimates. The corresponding identifiability scores discount the sensitivity measures by accounting for correlation between parameteric effects. Low scores indicate low potential for confidently identifying parameter values. In Yao et al. (2003), an identifiability cut-off value of 0.04 was recommended. All model parameters are identifiable by that criterion. (When model V7 was analyzed, the transconjugant growth rate was not above this identifiability threshold, and so it was eliminated to yield model V15.)

The last two columns of Table 2 contain estimates of 95% confidence intervals on the parameter estimates. These results, like the AIC-based model comparison, depend on an assumption that the errors are independent and normally distributed, which holds only approximately. The FIM-based estimate is derived under an assumption that the model itself is accurate (so that experimental error is the only source of variance). The complementary nonlinear regression approach incorporates model mismatch into its estimate. (A complementary upper bound calculation (Ashyraliyev et al., 2009) was not informative as it yielded estimates uniformally larger than ±100%.) While these results clearly indicate there is room for improvement, they suggest that there is strong potential for accurate estimation of the system kinetics with sufficient data.

Finally, Figure 3 demonstrates that the model can provide reasonably accurate predictions of system behavior. To reiterate, the data from the test experiments (#5–8) was not used for model fitting or model development. Moreover, the protocol followed for experiments #5–8 was dynamically distinct from the training experiments (#1–4). The training data (Figure 2) was collected from simultaneous loading experiments. The testing data (Figure 3) was collected from staggered loading experiments. Figure 3 thus demonstrates the model's ability to extrapolate to distinct dynamic regimes. The fits are not perfect, the worst prediction being the donor population in experiment #6 (perhaps as a result of the donor-recipient ratio at loading being too far beyond those of the training data). Nevertheless, the overall quality of the predictions speaks to the potential of the chosen model variant to predict system behavior.

### 4.3. Alternative modeling frameworks

Of course, any ODE-based model cannot be expected to provide highly accurate descriptions of surface-associated culture behavior, as spatial heterogenity is bound to have an effect on system dynamics. While the success of our approach indicates that such spatial features are not crucial to predicting behavior in the high-density mixed-distribution cultures observed in this study, spatial aspects will no doubt dominate in more heterogeneous environments or when subpopulations are not evenly distributed. Spatially explicit models offer appropriate frameworks in these cases: partial differential equation (PDE) models offer valuable descriptions of continuous spatial distributions, e.g., Massoudieh et al. (2010), while individual-based models provide increased resolution by describing the population in terms of individual cells (Kreft et al., 2013). Simple individual-based models represent cells as occupying positions in a regular lattice, e.g., Krone et al. (2007) and García and Rodríguez-Patón (2015), while more complex models, such as the DiSCUS model developed by Goñi-Moreno and Amos (2015) capture cell morphology. Related individual-based modeling frameworks, presented in Lardon et al. (2011) and Rudge et al. (2012), focus on biofilm formation but do not currently describe gene transfer. Importantly, these modeling frameworks have the capacity to capture inevitable variability within populations. Continued model developments will facilitate investigations of natural microbial populations (Marino et al., 2014; Widder et al., 2016) and the design of synthetic microbial ecologies (Zomorrodi and Segrè, 2016).

### 4.4. Conclusion

In this study we expanded on previous modeling efforts by assessing the quality of parameter estimates and the accuracy of extrapolative predictions. This project represents a step toward a modeling framework that can be used for confident prediction of the behavior of engineered microbial communities, such as those employed in the conjugation-based cellular computing systems described in Goñi-Moreno et al. (2013). In particular, plasmid-mediated bioaugmentation holds promise in a range of applications, including bioremediation (Top et al., 2002) and suppression of antibiotic resistance (Baquero et al., 2011; Gooding-Townsend et al., 2015) (see also Yosef et al., 2015). The success of such efforts will depend upon the development of techniques for accurate model-based design in cellular bioengineering.

## Author contributions

Conceived and designed the study: BI, AM. Performed the experiments: AM, PS, AN. Analyzed the results: AM, AN, BI. Wrote and revised the manuscript: AM, AN, PS, BI.

## Funding

This research was supported by the Natural Sciences and Engineering Research Council (NSERC) of Canada, the Canada Foundation for Innovation (CFI) and a MITACS Globalink Fellowship to AM.

### Conflict of interest statement

The authors declare that the research was conducted in the absence of any commercial or financial relationships that could be construed as a potential conflict of interest.
